# Preparation of Wood Pivots

**Published:** 1849-07

**Authors:** J. Taft


### PREPARATION OF WOOD PIVOTS.

BY J. TAFT.

Though the demand for the operation of inserting pivot teeth is less frequent than formerly, yet it is desirable to make each part of the operation as nearly perfect as possible. To cast in a mite to promote this object is the design of these lines.

The wood for pivots should be the best hickory; that from a small tree of slow growth I think preferable, and after being well seasoned—and this should not be rapidly done—it is best prepared in the following manner.

Take the blocks from six to eight inches long, and split them into rods about one fourth of an inch square, then with knife and file dress them down till they are one-half or one-third larger than the pivots should be, then go to the draw bench, take the ordinary draw plate, and draw the rods, thus dressed through two or three holes of the draw plate, inverted, until each rod is of an equal thickness throughout its length, then turn the draw plate and draw the rods throught it in the same manner wire is drawn; continue to draw it down until all the pores of the wood are closed by the compression. They may be drawn so they will just fit into the holes of the artificial crowns; they can be made of different sizes by beginning with rods of various sizes; they should be slightly oiled before drawing through the plate.

There are several advantages obtained by preparing pivots in this manner; they are far more durable thus compressed, than the wood in its natural porous condition; and for two reasons there is more of the wood fibre contained in a given space, for this is pressed into and occupies the porous part. And again, the pores of the pivot being closed, there can be no place for deposite of vitiated secretions, which when deposited in the pivot would cause it rapidly to decay. Again, the compressed pivots are much easier inserted, both into the artificial crown, and fang, than the unprepared wood; it may be prepared so that it can be easily pressed into the crown and fang; into the crown without danger of cracking it, and into the fang without pressure or irritation. In a tender fang, much pain is experienced by pressing an unprepared pivot into it; with the compressed pivot this is avoided.

The compressed pivot is much more permanent, is much stiffer and stronger than the non-compressed. I have only given here what I consider to be facts, and these predicated upon the constant use of the compressed pivots for three years.

I know some object to the compressed pivots. All the objection that has even an apparent force is, that they are liable to too great expansion by the heat and moisture of the mouth; if this is an objection to the compressed pivots, it is an objection equally strong against the non-compressed, for the compressed pivots have as little tendency to expand as the non-compressed. The expansion of a pivot is no objection, while it is not so great as to split the fang or crown; and I have as often seen the fangs split with the non-compressed as with the compressed pivots; and more often will the artificial crown be broken with the former than the latter, for the compressed pivot is more easily and accurately fitted into the crown, can be pressed into the crown and fang with slight pressure, and retains its position permanently.

